# Evaluation of the Fungicidal Effect of Some Commercial Disinfectant and Sterilizer Agents Formulated as Soluble Liquid against *Sclerotium rolfsii* Infected Tomato Plant

**DOI:** 10.3390/plants11243542

**Published:** 2022-12-15

**Authors:** Rania A. A. Hussien, Mai M. A. Gnedy, Ali A. S. Sayed, Ahmed Bondok, Dalal Hussien M. Alkhalifah, Amr Elkelish, Moataz M. Tawfik

**Affiliations:** 1 Fungicide, Bactericide and Nematicide Department, Central Agricultural Pesticides Lab (CAPL), Agriculture Research Center (ARC), Giza 11835, Egypt; 2 Pesticide Formulation Research Department, Central Agricultural Pesticides Lab (CAPL), Agriculture Research Center (ARC), Giza 11835, Egypt; 3 Botany Department, Faculty of Agriculture, Fayoum University, Fayoum 63514, Egypt; 4 Department of Plant Pathology, Faculty of Agriculture, Ain Shams University, Cairo 11566, Egypt; 5 Department of Biology, College of Science, Princess Nourah bint Abdulrahman University, Riyadh 11671, Saudi Arabia; 6 Department of Biology, College of Science, Imam Mohammad Ibn Saud Islamic University (IMSIU), Riyadh 11623, Saudi Arabia; 7 Botany Department, Faculty of Science, Suez Canal University, Ismailia 41522, Egypt; 8 Botany Department, Faculty of Science, Port Said University, Port Said 42526, Egypt

**Keywords:** antifungal effect, chloroxylenol, phenic, phenol, *Sclerotium rolfsii*, tomato plants

## Abstract

Globally, root rot disease of tomato plants caused by *Sclerotium rolfsii* is a severe disease leading to the death of infected plants. The effect of some commercial antiseptics and disinfectant agents, such as chloroxylenol (10%), phenic (10%) and formulated phenol (7%) on the control of root rot pathogen and its impact on growth and chemical constituents of tomato seedlings cv. Castle Rock were investigated in vitro and in vivo. The antifungal activity was measured in vitro following the poisoned food technique at different concentrations of 1000, 2000, 3000 and 4000 µL/L. Disinfectant agents and atrio (80%) were tested in vivo by soaking 20-day-old tomato seedlings in four concentrations of 125, 250, 500 and 1000 µL/100 mL water for 5 min and thereafter planting in soil infested by *S. rolfsii*. Fresh and dry weight, shoot and root length, and chemical constituents of tomato seedlings infected by *S. rolfsii* were investigated at 35 days after planting (DAP). Experimental results indicated that chloroxylenol (10%) was the most effective on fungus in vitro, recorded an effective concentration (EC_50_ = 1347.74 µL/L) followed by phenic (10%) (EC_50_ = 1370.52 µL/L) and formulated phenol (7%) (EC_50_ = 1553.59 µL/L). In vivo, atrio (80%) and disinfectant agents at different concentrations significantly (*p* ≤ 0.05) reduced disease incidence, increased shoot and root lengths and increased dry and fresh weight. Additionally, it significantly increased chlorophyll a, chlorophyll b, total carotenoids, total carbohydrates, total proteins, and total phenols. The highest reduction of root rot incidence and increase tomato growth parameters, as well as chemical compositions, were recorded on tomato seedlings treated with atrio (80%) as well as formulated phenol (7%) at different concentrations, followed by chloroxylenol (10%) at 125 and 250 µL/100 mL, whereas phenic (10%) was found to be the least effective treatment. Therefore, the application of formulated phenol (7%) could be commercially used to control tomato root rot diseases and increase the quality and quantity of tomato plants since it is promising against the pathogen, safe, and less expensive than fungicides.

## 1. Introduction

Tomato (*Solanum lycopersicum* L.) is one of the most important vegetable crops in the world due to increasing demand, dietary value and widespread production [1]. It is a rich source of vitamins (A and C), minerals, beta-carotene, and a high amount of water [2]. The 10 leading producers of tomato in the world are China, India, Turkey, USA, Egypt, Italy, Iran, Spain, Mexico and Brazil [3]. The world’s tomato production in 2020 was 187 million tonnes, with an average yield of 37 tonnes per hectare [3].

*Sclerotium rolfsii* is a destructive disease for many plants, causing damping-off and root rot of nursery seedlings, wilting and blight on adult plants [4]. It is responsible for high economic losses sustained by tomato producers each year [5]. Chemical fungicides have been used for disease management, become an integral part of agriculture, and increased food production [6]. However, they are costly; their extensive use has raised concerns about residual effects and toxicity to humans, animals and the environment, and efficacy has also decreased due to the emergence of fungicide-resistant pathogens [7].

On the contrary, antiseptics and disinfectants are non-selective and anti-infective agents that can be applied topically. They are used in healthy sectors and care centers to inhibit the growth of microbes and inanimate objects [8]. Chloroxylenol or para-chloro-meta-xylenol (PCMX) is a commercially cheap liquid antiseptic with low toxicity, low metal corrosivity, an active pH of 4–9, and is yet powerful enough to use as a disinfectant due to its broad spectrum of antimicrobial effects, that is, bacteria, fungi and yeast. It can kill 98% of microbes in just 15 s [9].

Phenol (carbolic acid) is generally a protoplasmic poison and was the first antiseptic employed by Joseph Lister (1912–1927), and displays effective antimicrobial activity against a wide range of microorganisms. Recently, phenols have been widely used in the healthcare, cosmetic, food and pharmaceutical industries to prevent unwanted microbial resistance [10]. It is less potent than chloroxylenol in inhibiting microorganism activity [11].

This research aims to find a new safe characteristic of the active ingredient and formulate it in a suitable formulation type to be used as an alternative to conventional fungicides. Additionally, we determine the antifungal activity of some commercial antiseptics and disinfectant agents and recommend the best agent to control tomato root rot disease caused by *S. rolfsii*.

## 2. Results

### 2.1. Characterization of Formulation Components

The total solubility of phenol is 100%, 62.5% and 33% in acetone, xylene and water, respectively. It exhibits acidic properties as seen by its free acidity of 0.098, suggesting that it requires an acidic adjuvant for its formulation (Table 1).

Data in Table 2 show the physico-chemical properties of surfactants, that is, Sisi 6, polyethylene glycol 600 mono laurate (PEG 600 ML), and polyethylene glycol 600 di-laurate (PEG 600 DL) used for preparing phenol as soluble concentrates. According to the hydrophilic–lipophilic balance (HLB), Sisi 6 and PEG 600 ML dispersed agents since their HLB values were more than 13, whereas PEG 600 DL was ~10–12. On the other hand, these surfactants reduced the surface tension values compared to water. The critical micelle concentration (CMC) of Sisi 6 was 0.5% and possessed low surface tension of 28.5 dyne/cm, and the CMC value of PEG 600 ML was 0.3% and possessed low surface tension of 30.64 dyne/cm, and the CMC value of PEG 600 DL was 0.4% and possessed low surface tension of 30.23 dyne/cm. Further, the acidic properties were 0.245, 0.882, and 0.049 for Sisi 6, PEG 600 ML, and PEG 600 DL, respectively. These findings conclude that all surface-active agents tested were suitable for preparing phenol as soluble concentrate formulation. Since the HLB of a surfactant is related to its solubility, a surfactant having a high range (13) will tend to be water-soluble [12].

CMC is important in the selection of surfactants for specific applications. Generally, at a concentration greater than the CMC value, the surface tension of the solution does not decrease with a further increase in surface tension concentrations [13]. In addition, the solubility of surfactant in water is considered an approximate guide to its HLB [14]. These findings conclude that the surfactants were suitable as a spreading agent to prepare the soluble liquid formulation.

Data in Table 3 demonstrate the physicochemical properties of the commercial disinfectants before and after storage at (54 ± 3 °C) for three days. Free acidity, alkalinity, and surface tension of formulated phenol (7%) and phenic (10%) did not change. In contrast, a slight decrease in free alkalinity was shown in chloroxylenol after three days of storage. Further, these disinfectants were soluble and clear with no sedimentation in both cases, indicating the ability of the formulation to keep its properties either before or after storage conditions [13].

### 2.2. Physicochemical Properties of Spray Solution at the Recommended Field Dilution Rate (1.5%)

Phenol-formulated and other commercial disinfectants showed low surface tension values, high viscosity, high electrical conductivity, and a low alkaline pH value compared to water and the active ingredient (Table 4). The surface tension (dyne/cm) of the spray solution was 28, 33.44, and 34.58 recorded in formulated phenol (7%), phenic (10%), and chloroxylenol (10%), respectively. The pH of the spray solution was 7.42, 8.46, and 8.94 recorded in formulated phenol (7%), chloroxylenol (10%), and phenic (10%), respectively. The viscosity (cm/poise) of the spray solution was 1.71, 1.70, and 1.20 in formulated phenol (7%), phenic (10%), and chloroxylenol (10%), respectively. Further, the conductivity (µMHOS) of the spray solution was 585, 448, and 425 in phenic (10%), chloroxylenol (10%), and formulated phenol (7%), respectively.

### 2.3. The Antifungal Activity of Disinfectant and Atrio (80%) on S. Rolfsii by Poisoned Food Technique In Vitro

**Figure 1:**
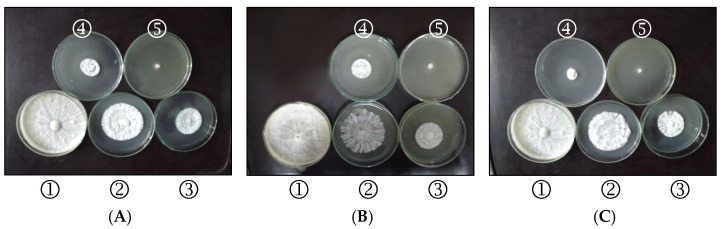
The antifungal activity of disinfectant agents in vitro measured by poisoned food technique. (**A**) Formulated phenol (7%); (**B**) chloroxylenol (10%); (**C**) phenic (10%). ➀ Control, ➁ 1000 µL/L, ➂ 2000 µL/L, ➃ 3000 µL/L, and ➄ 4000 µL/L.

Data in Table 5 and Figure 1 representing the application of disinfectant agents, that is, formulated phenol (7%), chloroxylenol (10%), and phenic (10%) at four concentrations of 1000, 2000, 3000, and 4000 µL/L show induced reduction on linear growth of *S. rolfsii* in vitro. No fungus growth was observed at a high concentration of all compounds. Further, the lowest EC50 value was observed by chloroxylenol (10%) (1347.74 µL/L) followed by 10% phenic (1370.52 µL/L) and 7% formulated phenol (1553.59 µL/L).

### 2.4. The Antifungal Activity of Disinfectant and Atrio (80%) on S. rolfsii (Greenhouse Conditions)

#### 2.4.1. Effect of disinfectant agents at different concentrations and Atrio 80% on the incidence of tomato root rots caused by *S. rolfsii* at 35 DAP (greenhouse conditions)

Tomato seedlings planted in soil infected with *S. rolfsii* showed disease symptoms (yellowing, root rot and crown rot). The disease incidence was 93.75% at 35 DAP compared to control sterilized soil. Tomato seedlings treated with a high concentration (1%) of formulated phenol (7%) as well as atrio 80%, recorded lower disease incidence ~12.50%, whereas the concentrations 0.5% and 0.25% of formulated phenol (7%) recorded ~18.75% and 25.0%, respectively. Therefore, treating tomato seedlings with formulated phenol (7%) at different concentrations of 1, 0.5, and 0.25% caused disease reduction of ~86.7, 80.0, and 73.3%, respectively, compared to other tested agents.

**Figure 2:**
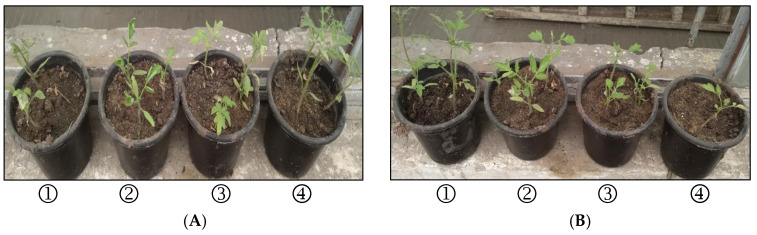

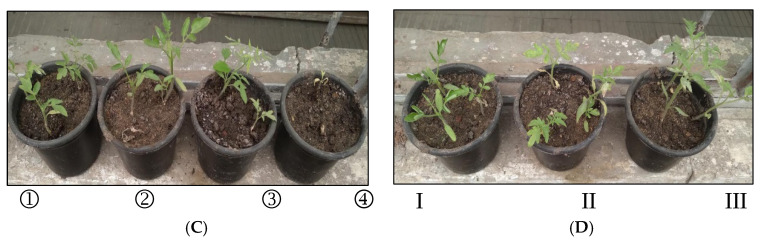
Effect of disinfectant agents at different concentrations and atrio 80% on the incidence of tomato root rots caused by *S. rolfsii* at 35 DAP (greenhouse conditions). (**A**) Formulated phenol (7%); (**B**) chloroxylenol (10%); (**C**) phenic (10%); (**D**) (**I**) Atrio (80%); (**II**) control pathogen; (**III**) healthy control. ➀ 125 µL 100 mL^−1^; ➁ 250 µL 100 mL^−1^; ➂ 500 µL 100 mL^−1^; ➃ 1000 µL 100 mL^−1^.

The disease reduction recorded in seedlings treated with chloroxylenol (10%) at a concentration of 0.25% was 66.67%, whereas using phenic (10%) at a concentration of 0.125 reduced the disease by 53.33% (Table 6 and Figure 2). However, the high concentration (1%) of chloroxylenol and phenic antagonized the pathogen in vitro; it was toxic for the plant and killed the tomato seedlings after 5 days of treatment.

#### 2.4.2. Effect of Disinfectant Agents at Different Concentrations and Atrio 80% on Growth Parameters of Tomato Seedlings Infected by *S. rolfsii* at 35 DAP

**Figure 3:**
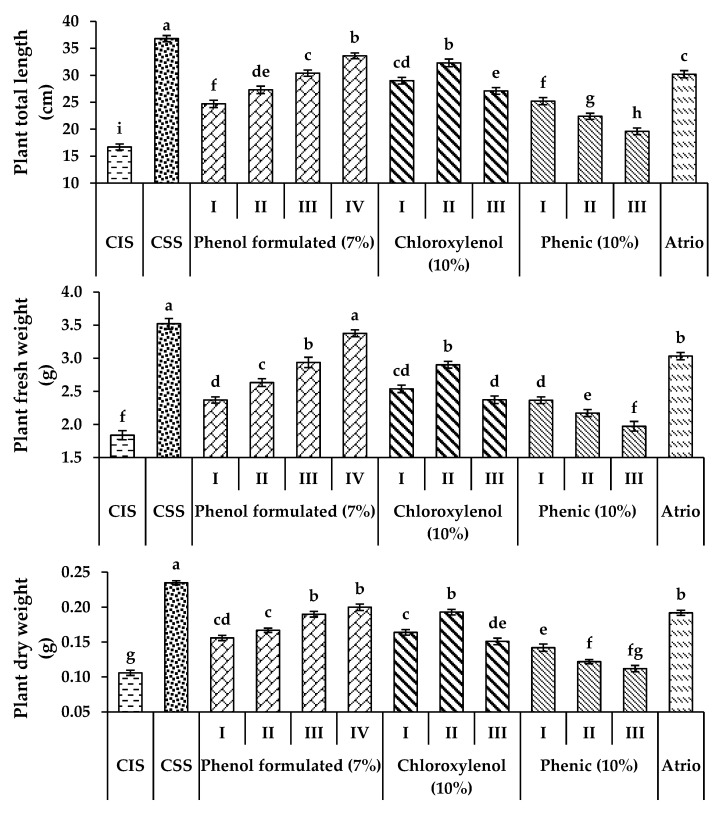
Effect of disinfectant agents at different concentrations and atrio 80% on growth parameters of tomato seedlings infected by *S. rolfsii* at 35 DAP. CIS; Control infected soil; CSS; control sterilized soil; (I) 125 µL 100 mL^−1^; (II) 250 µL 100 mL^−1^; (III) 500 µL 100 mL^−1^; (IV) 1000 µL 100 mL^−1^. The values shown in the figures are means ± SEM (*n* = 3), with different alphabetic letter/s being significantly different (*p* < 0.05) following Tukey’s post hoc test.

Tomato seedlings grown in soil infected with *S. rolfsii* recorded a reduction in all growth parameters, that is, plant height, shoot and root length, and fresh and dry weight at 35 DAP compared to the control sterilized soil (Figure 3, Table S1). Compared to untreated plants, tomato seedlings treated with atrio (80%) or disinfectant agents at different concentrations significantly (*p* ≤ 0.05) reduced tomato root rot incidence (Table 6) and increased all growth parameters at 35 DAP. The maximum increase in all growth parameters was recorded at a high concentration of 1% of formulated phenol (7%), atrio (80%), 0.5% of formulated phenol (7%), 0.25% of chloroxylenol (10%), and 0.125% of phenic (10%), respectively. The high concentration (1%) of chloroxylenol and phenic was toxic for the plant and killed the tomato seedlings after 5 days of treatment.

#### 2.4.3. Effect of Disinfectant Agents at Different Concentrations and Atrio 80% on Metabolite of Tomato Seedlings Infected by *S. rolfsii* at 35 DAP

**Figure 4:**
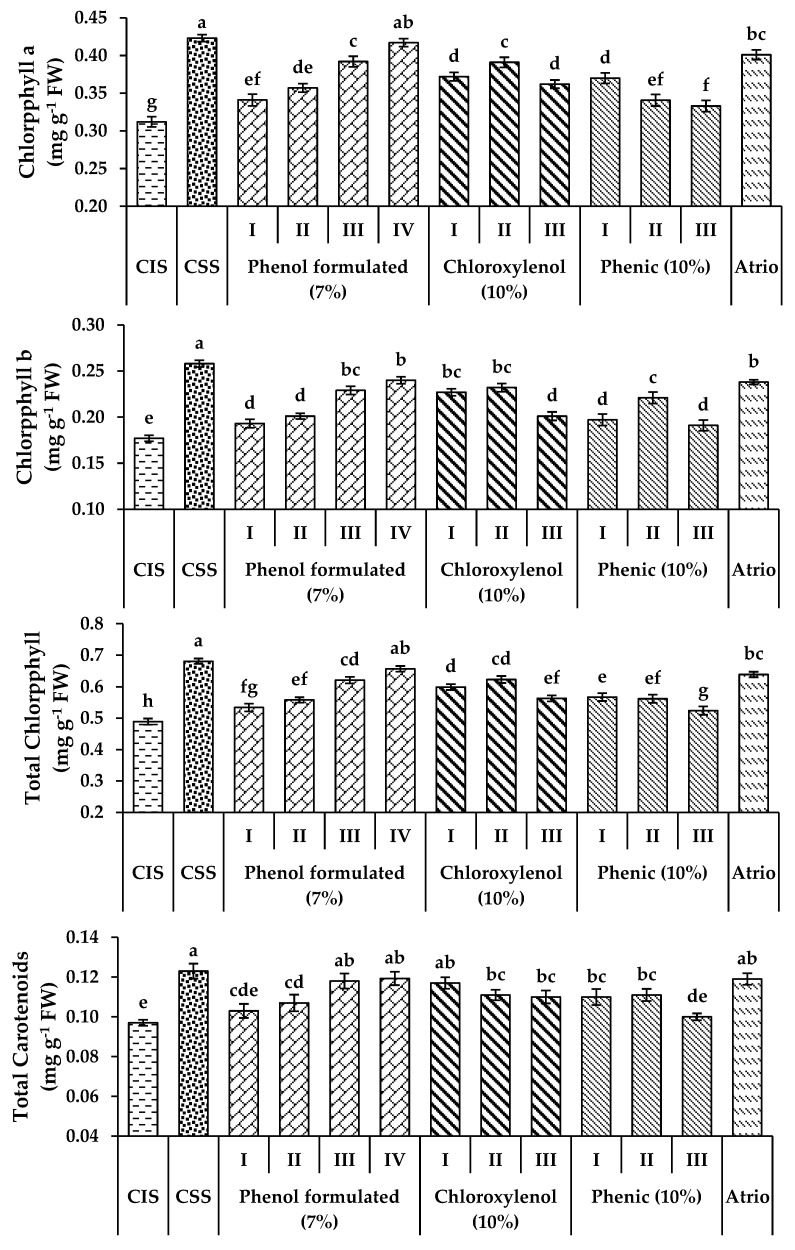

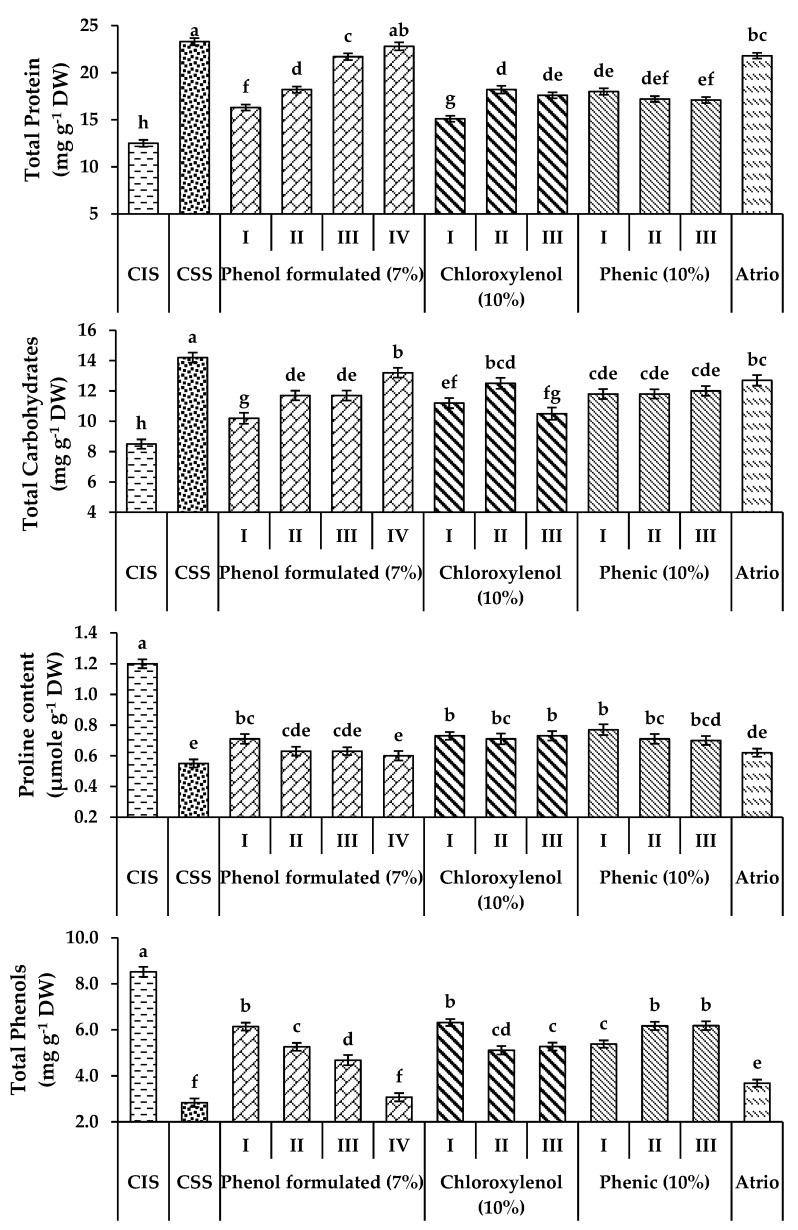
Effect of disinfectant agents at different concentrations and atrio 80% on chemical constituents of tomato seedlings infected by *S. rolfsii* at 35 DAP. **CIS**; Control infected soil; CSS; control sterilized soil; (I) 125 µL 100 mL^−1^; (II) 250 µL 100 mL^−1^; (III) 500 µL 100 mL^−1^; (IV) 1000 µL 100 mL^−1^. The values shown in the figures are means ± SEM (*n* = 3), with different alphabetic letter/s being significantly different (*p* < 0.05) following Tukey’s post hoc test.

Compared to control sterilized soil, tomato seedlings planted in soil infected with *S. rolfsii* recorded low leaf pigment concentration (Figure 4, Table S2). Tomato seedlings treated with atrio (80%) and disinfectant agents at different concentrations significantly (*p* ≤ 0.05) reduced tomato root rot incidence (Table 6) and increased chlorophyll A and B, total chlorophyll, and total carotenoids concentration in leaves of tomato seedlings at 35 DAP compared to untreated plants. The maximum increase in leaf pigments was recorded at a 1% concentration of formulated phenol (7%), atrio (80%), 0.5% of formulated phenol (7%), 0.25% of chloroxylenol (10%), and 0.125% of phenic (10%), respectively.

Further, treating with atrio (80%) and disinfectant agents at different concentrations significantly (*p* ≤ 0.05) improved chemical constituents, that is, total carbohydrates, protein content, proline content and total phenols of tomato seedlings at 35 DAP compared to untreated plants. The maximum increase in chemical constituents of tomato seedlings was recorded at a 1% concentration of formulated phenol (7%), atrio (80%), 0.5% of formulated phenol (7%), 0.25% of chloroxylenol (10%), and 0.125% of phenic (10%), respectively (Figure 4, Table S3). The high concentration (1%) of chloroxylenol and phenic was toxic for the plant and killed the tomato seedlings after 5 days of treatment.

## 3. Discussion

The tomato is an important vegetable crop due to its economic importance and nutritional value. It is one of the most important crops in Egypt and is used for food and industrial purposes [15]. *Sclerotium rolfsii* is a necrotrophic soil-borne plant pathogen that attacks more than 500 plant species belonging to over 100 families, causing chlorosis and wilting of entire plants and finally reducing crop yield and quality [16,17]. Synthetic fungicides have been used to control plant diseases worldwide. Although synthetic fungicides are highly effective, their repeated use has led to problems, such as environmental pollution, development of resistance, and residual toxicity [18].

The antiseptics used in this study are composed of phenols and chloroxylenol compounds. Each compound can be combined or used individually to achieve an antifungal effect. Disinfectants are used to treat the surface of inanimate objects and eliminate all pathogenic microorganisms by causing the denaturation of microbial proteins or enzymes. The antimicrobial properties of the disinfectant against some pathogenic bacteria have been reported earlier [19].

The physicochemical properties of phenol were carried out to determine the appropriate formulation type (Table 1). When selecting a pesticide formulation, several factors must be addressed; that is, the feasibility of utilizing a certain formulation in a specific location to control the target pest and whether the created product will offer effective control [20]. According to the Ref. [21], the pesticide which can be formulated is limited by solubility and hydrolytics. Therefore, the soluble liquid formulation is suitable for the tested material.

Results showed that surfactants applied to soluble liquid would reduce the surface tension of spray droplets, providing more coverage for toxicants by decreasing the contact angle of the spray on a solid surface [22]. The active ingredient with various inert components, known as additives or adjuvants, enhances the active ingredient’s effectiveness. Spreaders, wetting agents, stickers, foaming agents, and compatibility agents are all common additives [23]. Phenol showed free acidity, and one of the proposed surfactants (Sisi 6, PEG 600 ML, and PEG 600 DL) also showed free acidity, indicating that it might be employed in the formulation procedure of this chemical with no chemical interaction expected [23].

There were no observable changes for the soluble concentrate local formulation and commercial disinfectants before and after accelerated storage (Table 3), as it showed nearly the same values for free acidity or alkalinity, surface tension and solubility with no sedimentation in both cases, indicating the ability of the formulation to keep its properties in either normal or accelerated storage conditions with expected stability [24].

The spray solution used at a dilution rate (1.5%) had low surface tension, high viscosity, high conductivity, and low pH. Lowering the surface tension of a pesticide spray solution predicts increased wettability and spreading over the treated surface, which leads to increased pesticidal efficiency [25]. Increasing spray solution viscosity causes less drift and increases retention, sticking, and pesticidal effectiveness [26]. Increasing the electrical conductivity of the spray solution would lead to the deionization of insecticides and increase their deposit and penetration on the tested surface, resulting in an increase in insecticidal efficiency [27].

Application of selected disinfectant agents, that is, chloroxylenol (10%), phenic (10%) and formulated phenol (7%) at four concentrations 1000, 2000, 3000 and 4000 µL/L showed induced reduction in linear growth of *Sclerotium rolfsii* in vitro. Our results were in line with an earlier study [28] reporting that chloroxylenol had complete antifungal activity. Phenol and chloroxylenol cause the denaturation of proteins and inhibition of enzymes in microorganisms [29,30].

In this study, treating tomato seedlings with different disinfectant agents led to disease reduction, explaining the improvement of plant growth parameters, photosynthetic pigments and chemical constituents compared to those which were untreated [31]. However, there are no adequate studies on the effect of antiseptic and disinfectant agents against phytopathogenic fungi.

## 4. Materials and Methods

### 4.1. Tested Materials

#### 4.1.1. Commercial Disinfectants

- Chloroxylenol, or para-chloro-meta-xylenol (PCMX), is a mixture of 4.8% chloroxylenol + 9.9% terpineol and absolute alcohol. It was supplied by Agricultural Development Markets, Nadi El Seid St., Dokki, Giza.
- Phenic contains more than 98% high-quality, high-impact saponified tar oils and carbonates. It has between 6.5 and 7% pure phenol, a highly effective disinfectant. It is produced by the International Company for Chemicals and Industrial Detergents (Cairo, Egypt).

#### 4.1.2. Active Ingredient

Phenol or carbolic acid (C_6_H_5_OH), a white crystalline solid, was supplied by EL-Gomhoria Co., Cairo, Egypt.

#### 4.1.3. Surface-Active Agents

- Sisi-6, an anionic surfactant prepared by neutralizing aryl alkyl sulphonic acid with alkaline.
- Polyethylene glycol 600 di-laurate (PEG 600 DL) (Alexandria, Egypt), a nonionic surfactant supplied by The National Company for Starch, Yeast and Detergents, Alexandria.
- Polyethylene glycol 600 mono laurate (PEG 600 ML), a nonionic surfactant, supplied by The National Company for Starch, Yeast and Detergents, Alexandria.

### 4.2. Physico-Chemical Properties of Basic Formulation Constituents

#### 4.2.1. Active Ingredient

- Solubility is determined by measuring the volume of distilled water, acetone and xylene for complete solubility or miscibility of one gram of an active ingredient at 20°C [32]. The solubility (%) was calculated according to the following equation:
  (1) $$\mathrm{Solubility}(\%)=\frac{W}{V}\times 100$$
  W = Active ingredient weight, V = Volume of solvent required for complete solubility.
- Free acidity or alkalinity was determined according to the Refs. [33,34].

#### 4.2.2. Surface-Active Agents

- Surface tension was measured using a Du-Nouy tensiometer for solutions containing a 0.5% (*w*/*v*) surface-active agent following the American Society of Testing Materials [35].
- Hydrophilic–lipophilic balance (HLB): The solubility of a surfactant in water was used to approximate its hydrophilic–lipophilic balance [14].
- Critical micelle concentration (CMC): The concentration of the tested surfactants at which the surface tension of the solution does not decrease as the surfactant concentration increases (CMC) was determined using the technique given by the Ref. [13].
- Free-acidity or alkalinity was determined as mentioned previously.

### 4.3. Preparation of Phenol as Soluble Concentrate Formulation

The formulation of phenol was carried out by combining an active ingredient and surfactant in water in three forms (7% + 5% + 88%), (7% + 7.5% + 85.5%), and (7% + 10% + 83%). These three mixes were exposed to several tests to determine the optimal composition. The surface tension of the prepared mixtures was then evaluated at a field dilution rate of (0.5%), and the combination with the lowest surface tension was regarded as successful since it demonstrated the best wetting, spreading, and pesticidal efficiency when sprayed over the treated surface.

### 4.4. Physicochemical Properties of Disinfectants before and after Storage

- Surface tension was determined as mentioned before.
- Free acidity or alkalinity was determined as mentioned previously.
- Accelerated storage was done to check the stability of local formulations at 54 ± 30 °C for three days according to the Ref. [35].

### 4.5. Determination of the Physico-Chemical Properties of the Spray Solution at the Field Dilution Rate

- Surface tension was measured using the du Nouy Tensiometer method described by the Ref. [36].
- The pH was determined using an Adwa (AD8000) pH meter [35].
- Viscosity was measured at room temperature with a “Brookfield DV II + PRO” digital viscometer and UL rotational adaptor (ULA) [37].
- Electrical Conductivity was measured using Cole–Parmer pH/Conductivity following the method described by the Ref. [35].

### 4.6. Isolation and Identification of the Fungal Pathogen

Tomato plants showing typical root and crown rot symptoms were collected from a private field in Qaliubia Governorate, Egypt. The infected root samples were washed with tap water to remove the adhering soil particles, cut into small fragments, and surface-sterilized by dipping in 5% sodium hypochlorite solution for 5 min following the method [38]. The segments were washed several times with sterilized distilled water, dried between two folds of sterilized filter papers and transferred under aseptic conditions to sterilized Petri dishes containing potato dextrose agar medium (PDA). Thereafter, plates were incubated at 25 ± 2 °C, and developed colonies were picked up after five days, transferred onto a new PDA medium and purified using hyphal tip techniques [39]. The isolated fungus was identified microscopically according to Barnett and Hunter [40]. The identification was confirmed in the Plant Pathology Department, Faculty of Agriculture, Cairo University, Giza, Egypt. The stock culture was maintained on DPA slants and kept at 10 °C in the refrigerator for further experiments.

#### Antifungal Assay In Vitro

The antifungal activity of antiseptic and disinfectant agents, that is, chloroxylenol (10%), phenic (10%) and formulated phenol (7%) against *S. rolfsii* was investigated by using a food poisoning technique [41]. Disinfectant agents at concentrations of 1000, 2000, 3000, and 4000 µL/L were mixed with 50 mL of sterilized PDA medium and transferred equally into three Petri dishes. The media were allowed to solidify. Then a five-day old fungal culture disk of 9 mm diameter was taken and inoculated to the centre of the Petri dishes containing disinfectant agents. Instead of a PDA, a medium without disinfectant agents served as the control. All plates were incubated at 25 ± 2 °C, and radial growth of the colony was measured when the mycelia of control had almost filled the Petri dishes. Each test was performed in triplicate.

The fungal growth inhibition was calculated due to treatments against the control using the following formula [42]:

(2) $$\mathrm{Inhibition}(\%)=[\;\frac{C-T}{C}\times 100]$$

where C is the average of three replicates of hyphal extension (mm) of the control and T is the average of three replicates of hyphal extension (mm) of plates treated with tested material. EC_50_ and EC_90_ values were determined by the linear regression (LPD) line computer program of the tested fungus percentage inhibition probit vs. logs of the concentrations (µL/L) of the disinfectant’s agents. The EC_50_ and EC_90_ values indicate the effective concentrations (µL/L) that cause 50% and 90% growth inhibition. In essence, the lower the value of EC_50_ and EC_90_, the higher the efficacy of disinfectant agents in the test under consideration.

### 4.7. Greenhouse Experiment

Selected antiseptic and disinfectant agents, that is, chloroxylenol (10%), phenic (10%) and formulated phenol (7%), were evaluated for controlling root rot disease on tomato plants caused by *S. rolfsii* in the greenhouse (temperature (25 °C ± 2 °C) and humidity 65%) at the Central Agriculture Pesticide Laboratory, ARC, Giza, during the growing season 2021. Pots were filled with cornmeal sand medium at 3% (W/W), infested by *S. rolfsii* and watered regularly for 10 days before planting to ensure the distribution of inoculum. Tomato seedlings cv. Castle rock of 20 days old were soaked in the tested materials at different concentrations of 125, 250, 500, and 1000 µL/100 mL water for 5 min and fungicide (atrio 80% WP, Strchemi Industrial Chemicals, Egypt) at the recommended dose of 2 g L^−1^, individually. Tomato seedlings were planted at the rate of four seedlings/pot and four replicated (pots) were used for each treatment. At 35 DAP, root rot disease incidence was evaluated as the number of root-rot-diseased plants relative to the number of plant seedlings in each treatment according to the Ref. [43]. Disease incidence was calculated relative to the control infected soil. Fresh and dry weight and shoot and root length, growth parameters and chemical constituents of tomato seedlings infected by *S. rolfsii* at 35 DAP were determined as follows:

1. Total carbohydrates were determined and expressed as glucose according to the Shaffer–Somogi micro-method [44].
2. Total protein content was determined indirectly using nitrogen concentration estimated by the semi-micro-Kjeldahl method, and a Kjeldahl conversion coefficient of 6.25 was used [45].
3. Total phenols were determined using the colourimetric method of Folin–Denis as described by the Ref. [46].
4. Proline content was determined according to the method described by the Ref. [47].
5. Chlorophylls (a and b) and carotenoid concentrations were determined following the Ref. [48].

### 4.8. Statistical Analysis

The obtained data were subject to analysis of variance (ANOVA), using Minitab Statistical Software 20 [49]. Significant differences between means were compared at *p* < 0.05 following Tukey’s post hoc test.

## 5. Conclusions

Experimental results proved that treating tomato seedlings with high concentration (1%) of formulated phenol (7%) as well as low concentrations 0.25 and 0.5% of chloroxylenol (10%) improved growth parameters and chemical constituents of treated plants compared to those which were untreated. We also conclude that these disinfectants are more effective at controlling root rot disease; however, toxicological studies for the recommended agents are needed.

## Figures and Tables

**Table 1:** Physico-chemical properties of phenol as an active ingredient.

Solubility % (*w*/*v*)			Free Acidity as % H_2_SO_4_
Water	Acetone	Xylene	
33	100	62.5	0.098

**Table 2:** Physicochemical characteristics of surfactants used for preparing phenol as soluble concentrates.

Surface Active Agent	Surface Tension (dyne/cm) at CMC	CMC%	HLB	Free Acidity as % H_2_SO_4_
Sisi 6	28.5	0.5	>13	0.245
PEG 600 ML	30.64	0.3	>13	0.882
PEG 600 DL	30.23	0.4	10–12	0.049

**Table 3:** Physicochemical properties of disinfectants before and after storage at (54 ± 3 °C) for three days.

Storage	Physicochemical Properties	Commercial Disinfectants		
		Phenol Formulated	Chloroxylenol	Phenic
Before storage	Surface tension (dyne/cm)	40	36.97	36.97
	Free acidity as % H_2_SO_4_	0.249	0	0
	Free alkalinity as % NaOH	0.0	0.72	1.84
	Solubility	Soluble	Soluble	Soluble
	Sedimentation	nil	nil	nil
After storage	Surface tension (dyne/cm)	38	36.97	36
	Free acidity as % H_2_SO_4_	0.249	0	0
	Free alkalinity as % NaOH	0	0.52	1.84
	Solubility	Soluble	Soluble	Soluble
	Sedimentation	nil	nil	nil

**Table 4:** Physicochemical properties of spray solution at the recommended field dilution rate (1.5%).

Compounds	Physico-Chemical Properties			
	Surface Tension (dyne /cm)	*p*H Value	Conductivity (µMHOS)	Viscosity (cm/poise)
Water	72	9.21	350	0.89
Phenol	34.79	7.51	370	1.19
Formulated phenol (7%)	28	7.42	425	1.71
Chloroxylenol (10%)	34.58	8.46	448	1.20
Phenic (10%)	33.44	8.94	585	1.70

**Table 5:** Inhibitory effect (%) of antiseptic and disinfectant agents tested at different concentrations against *S. rolfsii* in vitro.

Compounds	Concentrations (µL/L)				EC_50_	EC_90_	Slope Value
	1000	2000	3000	4000			
Formulated phenol (7%)	33.33	55.55	72.22	100	3.1239 +/– 0.3266	3995.7593	1553.59
Chloroxylenol (10%)	36.66	64.44	88.88	100	3.2679 +/– 0.3469	3324.9652	1347.74
Phenic (10%)	38.88	58.88	85.55	100	3.123 +/– 0.3341	3525.7869	1370.52

**Table 6:** Effect of disinfectant agents at different concentrations and atrio 80% on the incidence of tomato root rots caused by *S. rolfsii* at 35 DAP (greenhouse conditions).

Treatments	Concentrations	Disease Incidence (%)	Disease Reduction (%)
Control infected soil	N/A *	93.75	0.00
Control sterilized soil	N/A	0.00	100.00
Formulated phenol (7%)	125µL 100 mL^−1^	43.75	53.33
	250µL 100 mL^−1^	25.00	73.33
	500µL 100 mL^−1^	18.75	80.00
	1000µL 100 mL^−1^	12.50	86.67
Chloroxylenol (10%)	125µL 100 mL^−1^	37.50	60.00
	250µL 100 mL^−1^	31.25	66.67
	500µL 100 mL^−1^	56.25	40.00
Phenic (10%)	125µL 100 mL^−1^	43.75	53.33
	250µL 100 mL^−1^	56.25	40.00
	500µL 100 mL^−1^	66.60	28.96
Atrio (80%)	2 g L^−1^	12.50	86.67

* N/A, not applicable.

## Data Availability

Date is contained within the article and supplementary material.

## Supplementary Materials

The following supporting information can be downloaded at: https://www.mdpi.com/article/10.3390/plants11243542/s1, Table S1. Effect of antiseptic and disinfectant agents at different concentrations on growth parameters of tomato seedlings infected by *S. rolfsii* at 35 DAP: Table S2. Effect of antiseptic and disinfectant agents at different concentrations on leaf pigments of tomato seedlings infected by *S. rolfsii* at 35 DAP: Table S3. Effect of antiseptic and disinfectant agents at different concentrations on chemical constituents of tomato seedlings infected by *S. rolfsii* at 35 DAP.
