# Supplementary material for: Evaluation of the Fungicidal Effect of Some Commercial Disinfectant and Sterilizer Agents Formulated as Soluble Liquid against Sclerotium rolfsii Infected Tomato Plant
Source: Plants (Basel). 2022 Dec 15;11(24):3542. doi: 10.3390/plants11243542 (PMC9784547; doi:10.3390/plants11243542)
Supplement: Supplementary file 1 [file plants-11-03542-s001.zip › plants-2039280-supplementary.pdf]

plants MDPI

**Supplementary Table S1.** Effect of antiseptic and disinfectant agents at different concentrations on growth parameters of tomato seedlings infected by *Sclerotium rolfsii* at 35 DAP.

| Treatments | | Root length (cm plant$^{-1}$) | | Shoot length (cm plant$^{-1}$) | | Total length (cm plant$^{-1}$) | | Fresh weight (g) | | Dry weight (g) | |
|---|---|---|---|---|---|---|---|---|---|---|---|
| **Disinfectants** | **Concentrations** | **Means ± SE** | **%** | **Means ± SE** | **%** | **Means ± SE** | **%** | **Means ± SE** | **%** | **Means ± SE** | **%** |
| Control infected soil | NA | 2.1±0.06$^{h}$ | 0 | 14.6±0.50$^{i}$ | 0 | 16.7±0.56$^{i}$ | 0 | 1.84±0.07$^{f}$ | 0 | 0.11±0.004$^{g}$ | 0 |
| Control sterilized soil | NA | 3.8±0.08$^{ab}$ | 81 | 33.0±0.55$^{a}$ | 126 | 36.8±0.58$^{a}$ | 120 | 3.52±0.08$^{a}$ | 92 | 0.24±0.003$^{a}$ | 122 |
| Phenol formulated (7%) | 125µl 100ml$^{-1}$ | 2.4±0.08$^{g}$ | 14 | 22.3±0.61$^{f}$ | 53 | 24.7±0.68$^{f}$ | 48 | 2.37±0.05$^{d}$ | 29 | 0.16±0.004$^{cd}$ | 47 |
| | 250µl 100ml$^{-1}$ | 2.7±0.09$^{ef}$ | 29 | 24.6±0.60$^{e}$ | 68 | 27.3±0.69$^{de}$ | 63 | 2.63±0.06$^{c}$ | 43 | 0.17±0.003$^{c}$ | 58 |
| | 500µl 100ml$^{-1}$ | 3.4±0.06$^{c}$ | 62 | 27.0±0.53$^{d}$ | 85 | 30.4±0.59$^{c}$ | 82 | 2.94±0.08$^{b}$ | 60 | 0.19±0.004$^{b}$ | 79 |
| | 1000µl 100ml$^{-1}$ | 4.0±0.09$^{a}$ | 90 | 29.6±0.46$^{b}$ | 103 | 33.6±0.54$^{b}$ | 101 | 3.38±0.05$^{a}$ | 84 | 0.20±0.005$^{b}$ | 89 |
| Chloroxylenol (10%) | 125µl 100ml$^{-1}$ | 2.8±0.06$^{e}$ | 33 | 26.2±0.56$^{de}$ | 79 | 29.0±0.61$^{cd}$ | 74 | 2.54±0.06$^{cd}$ | 38 | 0.16±0.004$^{c}$ | 55 |
| | 250µl 100ml$^{-1}$ | 3.6±0.06$^{bc}$ | 71 | 28.7±0.65$^{bc}$ | 97 | 32.3±0.71$^{b}$ | 93 | 2.90±0.05$^{b}$ | 58 | 0.19±0.004$^{b}$ | 82 |
| | 500µl 100ml$^{-1}$ | 2.5±0.09$^{fg}$ | 19 | 24.6±0.55$^{e}$ | 68 | 27.1±0.63$^{e}$ | 62 | 2.37±0.06$^{d}$ | 29 | 0.15±0.005$^{de}$ | 42 |
| Phenic (10%) | 125µl 100ml$^{-1}$ | 2.5±0.08$^{fg}$ | 19 | 22.7±0.59$^{f}$ | 55 | 25.2±0.66$^{f}$ | 51 | 2.37±0.05$^{d}$ | 29 | 0.14±0.005$^{e}$ | 34 |
| | 250µl 100ml$^{-1}$ | 2.4±0.07$^{g}$ | 14 | 20.0±0.50$^{g}$ | 37 | 22.4±0.57$^{g}$ | 34 | 2.17±0.05$^{e}$ | 18 | 0.12±0.003$^{f}$ | 15 |
| | 500µl 100ml$^{-1}$ | 2.3±0.08$^{gh}$ | 10 | 17.3±0.54$^{h}$ | 18 | 19.6±0.61$^{h}$ | 17 | 1.97±0.07$^{f}$ | 7 | 0.11±0.005$^{fg}$ | 6 |
| Atrio (80%) | 2 g L$^{-1}$ | 3.1±0.10$^{d}$ | 48 | 27.1±0.59$^{cd}$ | 86 | 30.2±0.67$^{c}$ | 81 | 3.03±0.05$^{b}$ | 65 | 0.19±0.004$^{b}$ | 81 |

The values showed in the table are means ± standard error, followed by different alphabetic letter/s are significantly different ($P < 0.05$) following Tukey's post-hoc test. Percent change (%) calculated based on control infected soil.

**Supplementary Table S2.** Effect of antiseptic and disinfectant agents at different concentrations on leaf pigments of tomato seedlings infected by *Sclerotium rolfsii* at 35 DAP.

| Treatments | | Chlorophyll a (mg $g^{-1}$ FW) | | Chlorophyll b (mg $g^{-1}$ FW) | | Total Chlorophyll (mg $g^{-1}$ FW) | | Total Carotenoids (mg $g^{-1}$ FW) | |
|---|---|---|---|---|---|---|---|---|---|
| **Disinfectants** | **Concentrations** | **Means ± SE** | **%** | **Means ± SE** | **%** | **Means ± SE** | **%** | **Means ± SE** | **%** |
| Control infected soil | NA | 0.31±0.007$^{g}$ | 0 | 0.18±0.003$^{e}$ | 0 | 0.49±0.010$^{h}$ | 0 | 0.097±0.002$^{e}$ | 0 |
| Control sterilized soil | NA | 0.42±0.005$^{a}$ | 36 | 0.26±0.004$^{a}$ | 46 | 0.68±0.008$^{a}$ | 39 | 0.123±0.004$^{a}$ | 27 |
| Phenol formulated (7%) | 125μl $100ml^{-1}$ | 0.34±0.008$^{ef}$ | 9 | 0.19±0.005$^{d}$ | 9 | 0.53±0.012$^{fg}$ | 9 | 0.103±0.004$^{cde}$ | 6 |
| | 250μl $100ml^{-1}$ | 0.36±0.006$^{de}$ | 14 | 0.20±0.003$^{d}$ | 14 | 0.56±0.009$^{ef}$ | 14 | 0.107±0.004$^{cd}$ | 10 |
| | 500μl $100ml^{-1}$ | 0.39±0.007$^{c}$ | 26 | 0.23±0.005$^{bc}$ | 29 | 0.62±0.010$^{cd}$ | 27 | 0.118±0.004$^{ab}$ | 22 |
| | 1000μl $100ml^{-1}$ | 0.42±0.005$^{ab}$ | 34 | 0.24±0.004$^{b}$ | 36 | 0.66±0.009$^{ab}$ | 34 | 0.119±0.003$^{ab}$ | 23 |
| Chloroxylenol (10%) | 125μl $100ml^{-1}$ | 0.37±0.006$^{d}$ | 19 | 0.23±0.004$^{bc}$ | 28 | 0.60±0.009$^{d}$ | 22 | 0.117±0.003$^{ab}$ | 21 |
| | 250μl $100ml^{-1}$ | 0.39±0.007$^{c}$ | 25 | 0.23±0.005$^{bc}$ | 31 | 0.62±0.011$^{cd}$ | 27 | 0.111±0.003$^{bc}$ | 14 |
| | 500μl $100ml^{-1}$ | 0.36±0.006$^{d}$ | 16 | 0.20±0.005$^{d}$ | 14 | 0.56±0.010$^{ef}$ | 15 | 0.110±0.003$^{bc}$ | 13 |
| Phenic (10%) | 125μl $100ml^{-1}$ | 0.37±0.007$^{d}$ | 19 | 0.20±0.006$^{d}$ | 11 | 0.57±0.013$^{e}$ | 16 | 0.110±0.004$^{bc}$ | 13 |
| | 250μl $100ml^{-1}$ | 0.34±0.008$^{ef}$ | 9 | 0.22±0.006$^{c}$ | 25 | 0.56±0.013$^{ef}$ | 15 | 0.111±0.003$^{bc}$ | 14 |
| | 500μl $100ml^{-1}$ | 0.33±0.008$^{f}$ | 7 | 0.19±0.006$^{d}$ | 8 | 0.52±0.013$^{g}$ | 7 | 0.100±0.002$^{de}$ | 3 |
| Atrio (80%) | 2 g $L^{-1}$ | 0.40±0.006$^{bc}$ | 29 | 0.24±0.003$^{b}$ | 34 | 0.64±0.009$^{bc}$ | 31 | 0.119±0.003$^{ab}$ | 23 |

The values showed in the table are means ± standard error, followed by different alphabetic letter/s are significantly different ($P < 0.05$) following Tukey's post-hoc test. Percent change (%) calculated based on control infected soil.

**Supplementary Table S3.** Effect of antiseptic and disinfectant agents at different concentrations on chemical constituents of tomato seedlings infected by *Sclerotium rolfsii* at 35 DAP.

| **Treatments** | | **Total Protein ($mg\ g^{-1}$ DW)** | | **Total Carbohydrates ($mg\ g^{-1}$ DW)** | | **Proline ($\mu mole\ g^{-1}$ DW)** | | **Total Phenols ($mg\ g^{-1}$ DW)** | |
|---|---|---|---|---|---|---|---|---|---|
| **Disinfectants** | **Concentrations** | **Means ± SE** | **%** | **Means ± SE** | **%** | **Means ± SE** | **%** | **Means ± SE** | **%** |
| Control infected soil | NA | 12.5±0.36$^{h}$ | 0 | 8.5±0.31$^{h}$ | 0 | 1.20±0.029$^{a}$ | 0 | 8.5±0.21$^{a}$ | 0 |
| Control sterilized soil | NA | 23.3±0.38$^{a}$ | 86 | 14.2±0.33$^{a}$ | 67 | 0.55±0.026$^{e}$ | -54 | 2.8±0.18$^{f}$ | -67 |
| Phenol formulated (7%) | 125µl 100ml$^{-1}$ | 16.3±0.31$^{f}$ | 30 | 10.2±0.36$^{g}$ | 20 | 0.71±0.032$^{bc}$ | -41 | 6.1±0.17$^{b}$ | -28 |
| | 250µl 100ml$^{-1}$ | 18.2±0.33$^{d}$ | 46 | 11.7±0.32$^{de}$ | 38 | 0.63±0.029$^{cde}$ | -48 | 5.3±0.17$^{c}$ | -38 |
| | 500µl 100ml$^{-1}$ | 21.7±0.35$^{c}$ | 74 | 11.7±0.33$^{de}$ | 38 | 0.63±0.025$^{cde}$ | -48 | 4.7±0.22$^{d}$ | -45 |
| | 1000µl 100ml$^{-1}$ | 22.8±0.42$^{ab}$ | 82 | 13.2±0.32$^{b}$ | 55 | 0.60±0.031$^{e}$ | -50 | 3.1±0.18$^{f}$ | -64 |
| Chloroxylenol (10%) | 125µl 100ml$^{-1}$ | 15.1±0.32$^{g}$ | 21 | 11.2±0.33$^{ef}$ | 32 | 0.73±0.025$^{b}$ | -39 | 6.3±0.15$^{b}$ | -26 |
| | 250µl 100ml$^{-1}$ | 18.2±0.42$^{d}$ | 46 | 12.5±0.36$^{bcd}$ | 47 | 0.71±0.035$^{bc}$ | -41 | 5.1±0.18$^{cd}$ | -40 |
| | 500µl 100ml$^{-1}$ | 17.6±0.32$^{de}$ | 41 | 10.5±0.40$^{fg}$ | 24 | 0.73±0.031$^{b}$ | -39 | 5.3±0.17$^{c}$ | -38 |
| Phenic (10%) | 125µl 100ml$^{-1}$ | 18.0±0.35$^{de}$ | 44 | 11.8±0.33$^{cde}$ | 39 | 0.77±0.035$^{b}$ | -36 | 5.4±0.16$^{c}$ | -37 |
| | 250µl 100ml$^{-1}$ | 17.2±0.31$^{def}$ | 38 | 11.8±0.31$^{cde}$ | 39 | 0.71±0.032$^{bc}$ | -41 | 6.2±0.18$^{b}$ | -28 |
| | 500µl 100ml$^{-1}$ | 17.1±0.32$^{ef}$ | 37 | 12.0±0.32$^{cde}$ | 41 | 0.70±0.029$^{bcd}$ | -42 | 6.2±0.19$^{b}$ | -27 |
| Atrio (80%) | 2 g L$^{-1}$ | 21.8±0.31$^{bc}$ | 74 | 12.7±0.35$^{bc}$ | 49 | 0.62±0.026$^{de}$ | -48 | 3.7±0.16$^{e}$ | -57 |

The values showed in the table are means ± standard error, followed by different alphabetic letter/s are significantly different ($P$ < 0.05) following Tukey's post-hoc test. Percent change (%) calculated based on control infected soil.
